# Iodine an Antidote to the Poison of the Curare

**Published:** 1854-04

**Authors:** 


*Iodine an Antidote to the Poison of the Curare.*

We find in a late number of the Gazette des Hopitaux the following which we are sure will be interesting to our readers.

Messrs Brainard and Green have communicated to the Academy (of Sciences,) a paper having the following title, On the use of Iodine an antidote to the poison of the Curare. One of the authors (M. Brainard,) has previously published the results of his experiments concerning the action of Iodine as an antidote to the bite of certain Crotali. In the new paper the experiments detailed go to show that the same solutions of Iodine have a similar influence over the American poison known as the Wooraro or Curare.

We have remarked, say our authors, a great analogy between the action of these poisonous preparations and the effect of the bites of of some of the American Serpents, In adition it has been well established that the venom of these serpents has at least something in common with these poisons; we have been led to experiment with the same remedies as antidotes, which we have previously found as efficacious against the bite of the serpents.

As they have found preparations of the Curare which differ in strength, the two authors have in the first place determined the activity of that which they made use of in their experiments and which had been given to them by M. the Prince Ch. Boneparte.

One Centigramme was sufficient to render a pigeon incapable of sustaining itself in 8 or 10 minutes, although it continued to live for an hour or more in a kind of lethargy, it was difficult to determine the exact moment of its death. It is neccessary to use but a very small quantity of the poison, otherwise its effects are so rapid that there is not time to have recourse to the antidote.

We have found that 2½ centigrammes are sufficient to kill a Guinea Pig in three minutes.

We mingled in a glass mortar five centigrammes of the poison triturating it with 20 drops of distilled water.

*Experiment first.*—We injected under the skin of a Guinea Pig, 10 drops of this solution, the operation was scarcely terminated before the animal fell on its side, and in about three minutes it was completely dead.

*Second Experiment*—We mingled 10 drops of this solution with 30 drops of an Iodine solution prepared as follows:

| | |
|---|---|
| Iodine - - - - | 0,50 |
| Iodid potass. - - - | 1,50 |
| Aquæ dist. - - - - - | 24,00 |

This mixture was kept for 30 minutes at the temperature of the body, and then injected under the skin of a Guinea Pig, the operation did not appear to effect the animal, the next morning it was apparently we.l.

*Third Experiment.*—As it might be objected that in our second experiment, the dilution of the poison in the water diminihsed its activity, we mixed 10 drops of the solution of Curare with 30 drops of distilled water, the whole was then injected under the skin of a Guinea Pig, it died at the end of three minutes.

*Fourth, Fifth, and Sixth Experiment.*—We mingled 12 drops of the solution of Cerare with 60 of the solution of Iodine; this mixture kept at the temperature of the body for 20 minutes, was divided into three equal parts, and injected under the skin of three pigeons, none of which were affected.

*Seventh Experiment.*—We mixed 4 drops of the solution of curare with 20 drops of the solution of Iodine, and injected the mixture immediately under the the skin of a pigeon which was not affected in the least.

As it might be supposed that the dilution of the poison in the water diminished its activity, we have assured ourselves that it is not so. This fact has been placed beyond doubt, by the results of the eighth, ninth and tenth experiments.

*Eleventh and Twelfth experiment.*—In order to place the subjects for experiment in the condition of those poisoned by a bite, we took two pigeons and injected under the skin of each 4 drops of the solution of Cerare, Immediately after we injected 20 drops of the Iodine solution in the wounds of each of the two pigeons, applying cups which were kept on five minutes. The two birds did not appear to suffer from this experiment.

*Thirteenth Experiment.*—After having shaved the flank of a Guinea pig, we injected under the skin 2½ centigrammes of the curare dissolved in 60 drops of water. Then we injected immediately the same quantity of the solution of Iodine; we applied cups, and the animal was not affected.

*Fourteenth Experiment.*—We injected 2½ centigrammes of the curare with 30 drops of water under the skin of a guinea pig, and submitted it to no treatment; it died in five minutes.

*Fifteenth Experiment.*—We injected under the skin of a pigeon 4 drops of the solution of curare, 30 drops of the solution of Iodine, and 30 drops of water; no effect was manifest.

*Sixteenth Experiment.*—We injected 4 drops of the solution of curare and 10 drops of water; the animal died in one hour.

*Seventeenth Experiment.*—We injected 5 drops of the solution of curare with 30 drops of the solution of Iodine and 30 drops of distilled water without any unpleasant effect.

*Eighteenth Experiment.*—In this experiment and the following we used a poison brought from the borders of the Amazon and known as the *ticunas.* This substance has the brilliant appearance of resin and a strong odor. It is known that it has been prepared less than four years. We used a solution of 5 centigrammes of the ticunas to 20 drops of water; 5 drops of this solution injected under the skin of a pigeon produced death in seven minutes.

*Nineteenth Experiment.*—We injected 8 drops of this solution of ticunas and two minutes after 20 drops of the Iodine solution and 20 drops of distilled water; we applied cups for 3 minutes. No effect was manifested.

*Twentieth Experiment.* We injected 8 drops of the solution of ticunas; then 20 drops of the Iodine solution; no effect was produced.

We believe ourselves justified in drawing from the preceding facts the following conclusions:

1st. The solution of Iodine and Iodide of Potassium is within certain limits a perfect antidote to the curare; mixed with it (as in solution) it destroys its poisonous effect.

2d. The Iodine solution injected immediately after the solution of curare neutralises completely its effects, provided that care be taken to apply a cup in order to arrest the circulation, and thus allow the Iodine to come in contact with the poison. It produces neither suppuration nor loss of substance by gangrene.

3d. The Iodine solution applied upon the surface of a deep wound of the muscles, in which the curare has been introduced, prevents the effects of the poison.

4th. The solution of Iodine has upon the curare an action perfectly identical with that which we have previously shown it to possess over the poison of the crotalus (Mem. presented to the academy Nov. 28th, 1843.)

5th. The identity of the effects of the curare and the poison of the crotalus, their same odor, and the effect of Iodine in controlling their actions, give much weight to the opinion already sufficiently indicated, that the active principle of the curare and analogous preparations is identical with that of the crotalus preserved in a particular manner.

---

*Iodine—an Antidote to the poison of the Rattlesnake.*

